# Sex Hormone-Binding Globulin and Metabolic Syndrome in Children and Adolescents: A Focus on Puberty

**DOI:** 10.3390/metabo15080494

**Published:** 2025-07-22

**Authors:** Banu Aydin, Stephen J. Winters

**Affiliations:** 1 Department of Medical Cell Biology, Uppsala University, 751 23 Uppsala, Sweden; 2 Department of Women’s and Children’s Health, Uppsala University, 751 85 Uppsala, Sweden; 3 Division of Endocrinology, Metabolism and Diabetes, University of Louisville, Louisville, KY 40202, USA; stephen.winters@louisville.edu

**Keywords:** cardiovascular risk, insulin resistance, metabolic syndrome, obesity, pediatric health, puberty, sex hormone-binding globulin, type 2 diabetes

## Abstract

Metabolic syndrome (MetS) is a cluster of conditions, including obesity, insulin resistance (IR), dyslipidemia, and hypertension, that increase the risk of cardiovascular disease (CVD) and type 2 diabetes mellitus (T2DM). While studied often in adults, the increasing prevalence of MetS in children and adolescents underscores the need for its early detection and intervention. Among various biomarkers, sex hormone-binding globulin (SHBG) has gained substantial attention due to its associations with metabolic health and disease. This review provides a comprehensive overview of SHBG and its association with MetS, with a focus on the pediatric and adolescent population. The interplay between SHBG, puberty, and metabolic risk factors is explored, including racial and ethnic variations. SHBG plays a crucial role in transporting sex hormones and regulating their bioavailability and has been found to correlate inversely with obesity and IR, two key components of MetS. Puberty represents a critical period during which hormonal changes and metabolic shifts may further influence SHBG levels and metabolic health. Understanding SHBG’s role in early metabolic risk detection could provide novel insights into the prevention and management of MetS.

## 1. Introduction

Metabolic syndrome (MetS) is a cluster of conditions, including obesity, insulin resistance (IR), dyslipidemia, and hypertension, that increase the risk of developing cardiovascular disease (CVD) and type 2 diabetes mellitus (T2DM) [1]. MetS is not only a growing concern in adults but is also increasing in prevalence among children and adolescents, raising alarm for the need for early intervention and prevention strategies [1,2]. Among the various biomarkers and physiological regulators involved in MetS, sex hormone-binding globulin (SHBG) has gained attention for its strong associations with metabolic health and disease [3].

SHBG functions to transport sex hormones, such as testosterone and estradiol, in the bloodstream [4]. It binds these hormones with high affinity, thereby regulating their bioavailability, metabolic clearance rate, and access to target tissues [5,6]. In humans, SHBG levels are influenced by numerous physiological and pathological factors, including age, sex, race, hormonal status, and metabolic conditions [3]. Notably, low levels of SHBG are associated with obesity and IR, two key components of MetS [7]. Recent studies have suggested that SHBG might serve as a valuable biomarker for identifying individuals at risk for developing MetS, including pediatric populations for whom early detection is crucial for preventing long-term health consequences [3,8,9,10]. Finally, there is some evidence that SHBG may contribute directly to the development of MetS and T2DM [11].

Puberty is a critical period marked by significant hormonal changes that can influence metabolic health [12]. During puberty, the body undergoes numerous physiological changes, along with significant weight gain [13], and rapid changes in body composition [14]. While an appropriate amount of weight gain is necessary during pubertal development, excessive amounts may increase the risk for obesity, MetS, and CVD [15]. The interplay between SHBG levels and puberty is particularly important as SHBG levels decline during late childhood and puberty, coincident with increases in sex hormones and changes in body composition [3]. The interactions between puberty, MetS, and SHBG are complex and multifaceted [3].

This review aims to provide a comprehensive overview of the current understanding of SHBG and its association with MetS, with a particular emphasis on the period of puberty. We will review the biological functions of SHBG, its regulation, and the evidence linking SHBG levels to MetS components.

## 2. Overview of Sex Hormone-Binding Globulin (SHBG)

SHBG is a 90–100 KDa homodimeric glycoprotein that transports testosterone and other steroids in the circulation with high affinity (K_D_ ~1 nmol/L), reduces their metabolic clearance rate, and regulates their access to target tissues [5]. Human SHBG shows the highest binding affinity for DHT, followed by testosterone and then estradiol. It also binds medications such as levonorgestrel and fluoxymesterone [6]. The N-terminal domain of each subunit contains the steroid binding pocket as well as calcium and zinc binding sites which influence the affinity for steroid ligands and maintain the dimeric structure whereas the C-terminus contains sites for glycosylation [16,17]. There are one O-linked and two N-linked oligosaccharide chains on each of the SHBG monomers and variable glycosylation explains the variation in subunit molecular weight.

Circulating SHBG is produced primarily by hepatocytes of humans and various other mammals, yet it is reportedly undetectable in the plasma of adult rats, mice, guinea pigs, and pigs [18]. Although absent from adult rodent plasma, SHBG is expressed in fetal rat liver, and in Sertoli cells in rodents and other lower mammals where it is designated “androgen binding protein” [18]. A transcript expressed from an alternative upstream promoter with an alternative exon 1 is expressed in human testicular germ cells [19], and various alternately spliced transcripts are expressed at low levels in prostate, ovary, breast, liver, and brain [20].

**Figure 1:**
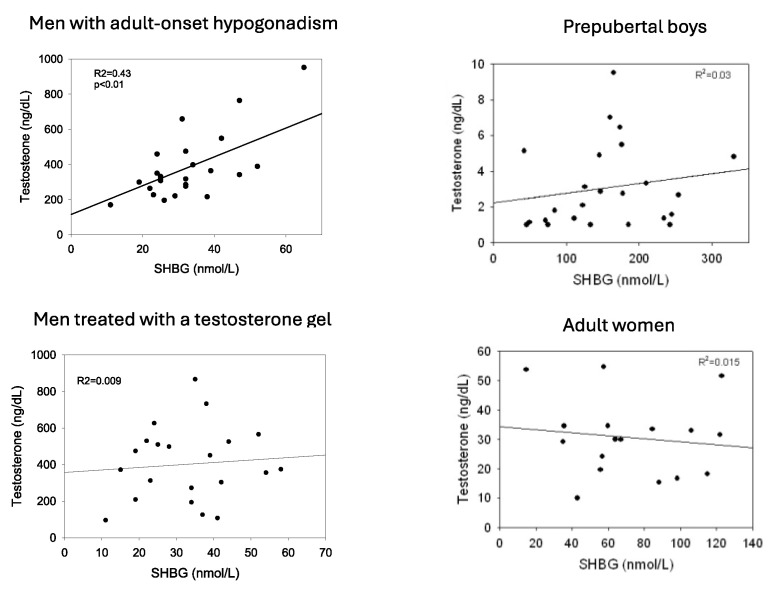
Relationship between plasma levels of SHBG and total testosterone in men with adult-onset hypogonadism, men with adult-onset hypogonadism receiving treatment with transdermal testosterone, prepubertal boys age 5–8, and normal adult cycling women. Redrawn in part from Winters [22].

Inasmuch as the SHBG-binding capacity generally exceeds the plasma testosterone concentration and binds testosterone with high affinity, the level of SHBG is one determinant of the total testosterone level [21]. Accordingly, men with low levels of SHBG (e.g., obesity) tend to have lower total testosterone levels whereas those with high levels of SHBG (e.g., hyperthyroidism) have a high total testosterone level. This association is strong in normal adult men [22] and in boys during minipuberty [23] but is modest in testosterone-treated men, prepubertal boys, and in women (Figure 1). One explanation for these variable relationships is androgen negative feedback regulation of gonadotropin secretion which is strong in adult men and in boys during minipuberty but is modest in prepubertal boys and in women and is disrupted in adult men by exogenous testosterone treatment. In the former, variable SHBG levels change the relative bound and free testosterone and estradiol concentrations, resulting in changes in LH and thereby testosterone secretion [22].

## 3. SHBG Gene Expression

The SHBG gene is located on human chromosome 17 p12–13 and is composed of 8 exons [24]. A small number of individuals with homozygous missense mutations in the SHBG coding region leading to a complete lack of plasma SHBG have been described. One such case involved an adult woman with undetectable plasma SHBG levels due to a compound SHBG heterozygote polymorphism [25]. During pregnancy, her initially mild hirsutism progressed dramatically to marked virilization, coinciding with a fourfold increase in her free testosterone concentration [25]. She delivered twin girls with no signs of virilization. This interesting case suggests that high levels of maternal SHBG during pregnancy protect the pregnant woman from placental hyperandrogenism. A separate report [26] described an adult male presenting with low total testosterone and undetectable SHBG, despite normal levels of LH and follicle-stimulating hormone (FSH). He reported symptoms including reduced libido, fewer spontaneous morning erections, fatigue, muscle weakness, low shaving frequency, along with small testes and a low bone density. Notably, his semen parameters were within the reference range. His sister, who was also affected, exhibited delayed onset of menstruation, underdeveloped breasts, and irregular menstrual cycles. These findings suggest that SHBG mediates androgen bioactivity. In vitro experiments revealed that the mutant SHBG was retained intracellularly. In another case report, gonadotropin-independent precocious puberty was reported in a 9 yo girl with non-detectable plasma SHBG and a homozygous polymorphism [27].

SHBG gene polymorphisms have also been reported that either increase or decrease the SHBG binding affinity for DHT and/or estradiol [28,29,30] and thereby the total testosterone level. An SNP rs6258 that reduces testosterone binding affinity and lowers total testosterone levels is present in 2% of European men [28]. There is also a point mutation in exon 8 that creates a consensus site for an additional carbohydrate chain that produces an SHBG with an extended half-life that was found in 17% of a French study population [31,32].

Certain SNPs of the SHBG gene are associated with the risk for adverse metabolic outcomes. Ding and colleagues [33] reported that SNPs, rs6257 and rs6259, are associated with a roughly 10% decrease or increase in circulating SHBG levels, respectively, and with a 68% increase or 38% decrease in the risk of developing T2DM by age 70. Perry et al. [34] conducted a meta-analysis in which carriers of the minor allele (A) of SNP rs1799941, which is just upstream of the proximal promoter of the SHBG gene and has been associated with SHBG levels in older men [35], was linked with elevated levels of SHBG and a lower risk of T2DM although other studies did not confirm this association [36]. A recent study of women and men in the Netherlands, most with overweight or obesity, reported that genetic variants associated with higher SHBG levels in other populations were associated with lesser amounts of liver fat [37]. A study of Turkish male and female children and adolescents found that the rs179441 polymorphism increased the risk for MetS three-fold [38].

There is also a polymorphic (TAAAA)n nucleotide repeat in the SHBG proximal promoter (rs5030991) that binds a 46 KDa protein that acts to regulate transcription. In HepG2 cells, fewer TAAAA repeats were associated with less SHBG transcriptional activity when compared with alleles of longer length [39]. On the other hand, there is a tendency to higher plasma SHBG levels in healthy young adult men with fewer TAAAA repeats [35,40]. Multiple studies have been performed in women with polycystic ovary syndrome (PCOS) in whom SHBG levels are low. While results have been inconsistent, a meta-analysis [41] found that polymorphisms of eight or more TAAAA repeats increase the risk for PCOS by 24%.

## 4. Ontogeny of SHBG Production

**Figure 2:**
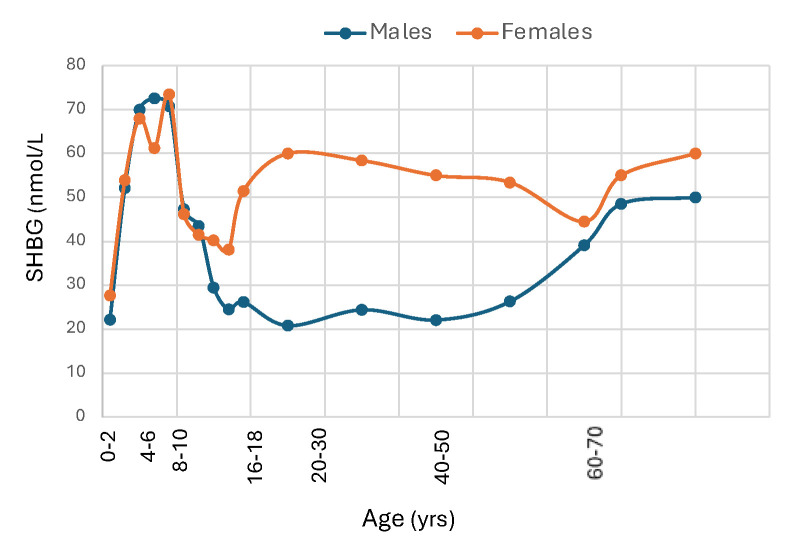
Median SHBG levels from birth to old age in male and female Caucasians. Redrawn from Elmlinger et al. [47], Burger et al. [58], Aribas E et al. [59].

The SHBG gene is expressed in the placenta [42]. SHBG is present in cord blood, which is of fetal origin, in which levels are much lower than in maternal blood, and most studies have found similar levels in males and females [43,44,45]. As illustrated in Figure 2, cross-sectional data suggest that SHBG concentrations increase markedly after birth into early childhood [46,47] and reach peak around 3 months of age [48]. While the explanation for this increase is uncertain, the concordant rise in T3 levels, which is known to stimulate SHBG production, and lower levels in hypothyroid infants [49] support the idea that the postnatal rise in SHBG is at least partly thyroid hormone-dependent. One idea is that the rise in SHBG serves to lower testosterone bioactivity in infant boys through childhood, as proposed by Hammond [5]. In support of this idea, the level of salivary testosterone, a surrogate for free testosterone, is much higher in newborn boys with lower SHBG levels than in minipuberty although total testosterone levels are similar [50,51]. During childhood, SHBG levels remain relatively high and stable, but begin to decline gradually [52] as puberty approaches, more prominently in boys than in girls [53], to reach nadir values in young adulthood [54]. Although the decline in boys is partly from androgens [55], a comparable decline in boys with idiopathic hypopituitarism [56] or with isolated hypogonadotropic hypogonadism or complete androgen insensitivity [57] suggests that metabolic rather than neuroendocrine signals are the main driver of the late childhood decline in SHBG.

Reference intervals for adults based on blood samples from 1477 healthy U.S. adults ages 40 and older that were collected in NHANES and analyzed by chemiluminescence assay were 12.6 to 92.4 nmol/L for men and 18.4 to 211.5 nmol/L for women [60]. However, SHBG levels are influenced by age and race, and may vary among assays. Adult women have higher SHBG levels than men partly due to stimulation by estrogens [61,62,63] while androgens produce a small decline in plasma SHBG [64]. Metabolic factors related to visceral adipose tissue in men versus gluteal-femoral adipose tissue in women may also contribute to the sex difference in SHBG concentrations. SHBG levels are higher in the luteal phase than in the follicular phase or at midcycle [65] and increase markedly during pregnancy [66]. SHBG levels in men rise gradually beyond age 60 [67] and while there is no significant change in women at menopause [58], levels rise slightly beyond age 65 [59,68,69].

## 5. Hormonal and Metabolic Regulation of SHBG Production

There is a 10- to 20-fold between-subject variation in serum SHBG levels beginning in newborns [45] through childhood [70] to adults [71] while the level of SHBG in a given healthy individual is relatively constant [72,73]. Studies of adult male twins have found a strong genetic component to circulating SHBG [74,75] and genome-wide association studies (GWAS) have identified single nucleotide polymorphisms (SNPs) in 60 loci that predict SHBG concentrations in adult men and women [76,77] and in newborns [78]. Similar genetic relationships in newborns and adult men suggest that adult SHBG levels are partly established in infancy. There is also a long list of hormones, metabolic factors, pathological disorders, and medications that are known to influence circulating SHBG levels (Table 1).

**Figure 3:**
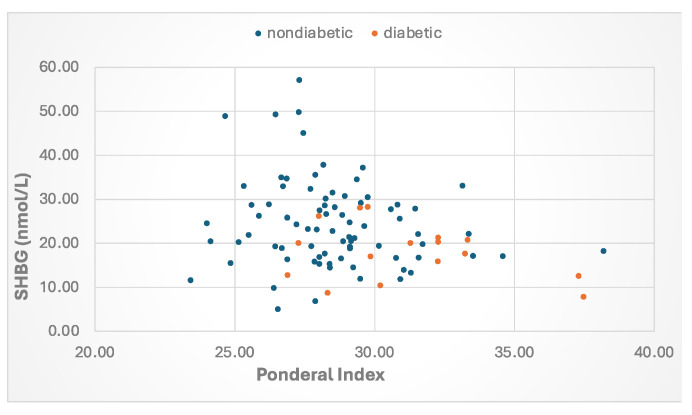
Relationship between the Ponderal index and SHBG levels at age day 2 in babies born to mothers with type 2 or gestational diabetes or those without diabetes. The Ponderal Index (PI) was calculated as BW (g) × 100/[BL (cm)]^3^. Data from Aydin et al. [45].

Beginning in newborns, metabolic factors are major determinants of plasma SHBG. Levels are lower among babies born to overweight mothers with either gestational diabetes [79] or T2DM [45]. and placental SHBG mRNA levels are reduced when mothers have gestational diabetes [80]. By postnatal day 2, there is an inverse correlation between the Ponderal index, a measure of newborn adipose tissue, and SHBG levels (Figure 3). Lower SHBG levels were found in prepubertal girls but not boys from Norway whose mothers had PCOS [81] and in South Asian-American boys and girls whose parents were diagnosed with MetS [8]. By the teenage years, SHBG levels are lower in both boys and girls who are overweight and diagnosed with MetS [82]. In adulthood, SHBG levels are lower with increasing obesity [83] and rise with weight loss [84,85] and are lower in men [86,87], middle aged [88], and postmenopausal women [89] who have features of MetS. Finally, results from NHANES 1988–91 versus 1999–2004 reveal that SHBG levels in adults across the U.S. have declined in recent years in parallel with increases in BMI and waist circumference [90].

Studies have found racial differences in circulating SHBG concentrations that may also be partly on a metabolic basis. There is a tendency to higher SHBG levels among individuals with African-American origin, including prepubertal boys [91], teenage girls with overweight or obesity and PCOS [92], glucose-intolerant post-menopausal women [93], and pre- and peri-menopausal African-American women than the general population [94]. On the other hand, SHBG levels in healthy young adult African-American women as well as Asian-American women were lower when compared to Caucasians in the Nurse’s Health Study [95]. Among Latinos, SHBG levels were lower in teenage Mexican-American boys [96], adult women [97], teenage girls [92], and adult Latino women with PCOS [98] than in Caucasians. SHBG levels are especially low in South Asians. Lower SHBG levels were reported in South Asian men compared to Europeans living in the U.K. [99], in women with PCOS seeking treatment for infertility in West Yorkshire, U.K. [100], and in healthy male and female adults in the western U.S. [101]. In the Boston Area Community Health survey, however, SHBG levels were similar among adult Caucasian, Black, and Hispanic men [102].

Birkeland et al. [103] were among the first to report that SHBG levels could serve as a marker of IR, a finding that has since been supported by numerous studies [104]. There is considerable evidence that the reduction in SHBG in IR is partly due to elevated insulin levels [105]. Several studies have demonstrated an inverse relationship between SHBG and insulin levels, whether measured in the fasting state [106], after glucose stimulation [107], or as 24 h insulin or C-peptide concentrations [108,109]. Notably, SHBG concentrations rise as IR improves and insulin levels decrease following weight loss [110], resistance exercise [111], or treatment with insulin-sensitizing drugs [112].

Experiments using HepG2 human hepatocarcinoma cells, which express the SHBG gene, have provided conflicting results. Early studies revealed that insulin suppressed SHBG production [113,114], and reduced SHBG mRNA expression [113]. These results were called into question, however, as later studies also using HepG2 cells found no effect of insulin on SHBG secretion or mRNA levels, which instead were reduced by adding glucose or fructose to the culture media [7] and by rosiglitazone [115]. These latter results are, however, at odds with the normal SHBG levels in patients with type 1 diabetes [116] who are also hyperglycemic, and with studies in patients with T2DM treated with rosiglitazone whose SHBG levels increased [117].

HNF4α is an orphan nuclear receptor that is a master regulator of hepatic development and function, including genes involved in triacylglycerol, cholesterol, and lipoprotein metabolism [118,119], and HNF4α is thought to play a central role in the development of fatty liver disease [120]. Functional HNF4α-binding sites have been identified in more than 140 genes, many of which are associated with the metabolism of glucose, lipids, and amino acids. There is an HNF4α binding site in the proximal promoter of the SHBG gene, and the transcriptional rate of an SHBG-luciferase reporter in HepG2 cells was enhanced by over-expression of HNF4α [121]. In a study of adult men and women undergoing liver resection for cancer, our group [63] reported a strong positive correlation between hepatic HNF4α and SHBG mRNA levels, and between SHBG mRNA in liver and circulating SHBG levels. Thus, HNF4α likely plays a key role in regulating SHBG.

Insulin resistance is characterized by a defect in insulin-stimulated glucose uptake in adipose and other tissues and leads to hyperinsulinemia. Xie et al. [122] showed that HNF4α mRNA in liver is suppressed in diabetic hyperinsulinemic db/db mice but not in mice rendered diabetic by streptozotocin-induced hypoinsulinemia, and that insulin inhibits hepatic HNF4α expression by stimulating transcription of SREBP. Insulin resistance is also associated with an increase in hepatic fat [123] and numerous studies have reported a strong inverse relationship between liver fat accumulation and circulating SHBG levels [124,125]. Furthermore, SHBG levels rise and liver fat decreases with weight loss [126]. In the aforementioned study of liver tissue from adult men and women undergoing liver resection for cancer [63], serum SHBG and SHBG mRNA levels were low in individuals with insulin resistance by HOMA or with elevated hepatic triglyceride concentrations. Low SHBG mRNA levels were not always explained by these factors, however, implying that other mechanisms must be operative. For example, larger studies are needed to clarify how sex, age, and race influence SHBG expression.

Increased hepatic fatty acids and lipotoxic metabolites increase production of cytokines, including TNF, IL-6, and IL-1b which initiate the production of pro-inflammatory signals including nuclear factor-κB (NF-κB) and c-jun n-terminal kinase (JNK) [127]. Selva and colleagues, using HepG2 hepatocarcinoma cells, showed that TNFα suppresses SHBG expression by decreasing HNF4α through a mechanism involving NF-κB [128], and that IL1β reduces SHBG mRNA through HNF4α via the MAPK kinase-1/2 and JNK signaling pathways [129].

**Figure 4:**
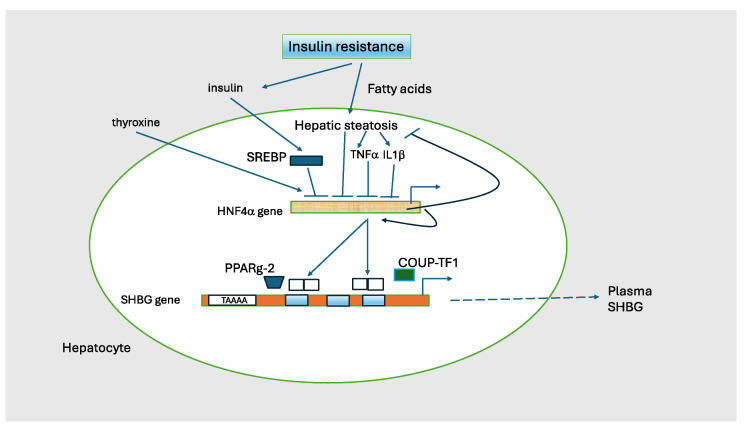
Regulation of SHBG transcription. The transcriptional activity of the SHBG gene is the major determinant of circulating SHBG concentrations. HNF4α is a nuclear transcription factor that activates multiple genes in liver including SHBG as a homodimer. It is the major on/off switch for SHBG. HNF4α is activated by thyroxine and is suppressed by insulin, TNF1, and 1L1b. HNF4α regulates lipid metabolism as knock-out mice develop severe steatosis. HNF4α also stimulates its own transcription. HNF4α can heterodimerize with COUP-TF1 (chicken ovalbumin upstream promoter transcription factor-1; also known as NR2F1) to elicit a transcription inhibitory signal. PPARg-2 (peroxisome proliferator-activated receptor gamma isoform 2) is a second nuclear hormone receptor that competes with HNF4α and represses SHBG transcription. The TAAAA sequence has a silencing activity that varies with the number of repeats. Adapted from Hammond GL [130].

Thus, as diagrammed in Figure 4, there is substantial evidence that hyperinsulinemia and excess hepatic fat are key determinants of low SHBG levels in patients with IR [130].

Reduced SHBG levels in patients with Cushing syndrome and those treated with glucocorticoids may be explained by IR [131]. For example, in children undergoing treatment with prednisone or dexamethasone for leukemia, SHBG levels gradually declined over a 4-week period, during which time both BMI and leptin levels increased [132]. Similarly, girls with congenital adrenal hyperplasia exhibit reduced SHBG levels [133], possibly due to central obesity and IR [134]. SHBG concentrations are also elevated in individuals with growth hormone deficiency [135], and are reduced in those with acromegaly [136], likely reflecting differences in insulin sensitivity and resistance, respectively.

Thyroid hormone directly increases SHBG production by activating HNF4α transcription [137]. SHBG levels are increased in hyperthyroidism in proportion to the levels of thyroxine (T4) and triiodothyronine (T3) in children [138] as in adults [139,140], and values normalize when hyperthyroxinemia is treated [141]. Resultant high levels of total testosterone may cause diagnostic confusion. On the other hand, SHBG levels are reduced in hypothyroidism [142], which in adult males can be misinterpreted as testosterone deficiency.

Liver diseases influence SHBG levels through multiple pathways. For example, individuals with alcoholic cirrhosis often exhibit elevated SHBG concentrations. This may result from alcohol-induced testicular damage which lowers testosterone levels and raises LH secretion. Elevated LH enhances testicular aromatase activity, increasing estradiol production, a known stimulator of SHBG synthesis. Additionally, stress-induced adrenal activation via ACTH can raise estrone and estradiol levels, further contributing to high SHBG [143]. Increased sulfatase activity (which converts inactive estrogen sulfates to active forms) may also play a role [144]. Hepatitis-B and C infections are similarly associated with substantial rises in SHBG [145]. Patients with liver disease caused by hemochromatosis typically develop hypogonadotropic hypogonadism from pituitary iron accumulation, often accompanied by mildly elevated SHBG. On the other hand, metabolic (non-alcoholic) fatty liver disease (MAFLD), characterized by hepatic triglyceride accumulation unrelated to excess alcohol consumption, is associated with increased visceral adipose tissue (VAT), IR, dyslipidemia and reduced SHBG levels [124,125,146]. Notably, low SHBG may serve as a predictive marker for the onset of MAFLD [147,148].

## 6. Direct Effects of SHBG

Some years ago, ligand-bound SHBG was reported to bind membrane receptors in prostate and other tissues and to stimulate cAMP production [149] to generate an intracellular effect. Since then, there is evidence that unliganded SHBG binds to the G-protein-coupled receptor GPRC6A and affects ERK 1/2 phosphorylation [150]. GPRC6A is expressed in testis, and null mice have smaller testes and seminal vesicles and reduced testosterone levels but are fertile [151]. Interestingly, these animals have increased hepatic triglyceride content, somewhat higher glucose levels, and reduced insulin sensitivity compared to w/t mice.

Unliganded SHBG was shown to inhibit inflammatory cytokine expression in mouse macrophages and decrease cytokines and transcription factors involved in adipogenesis and triglyceride synthesis independent of sex steroids in mouse-derived 3T3-L1 adipocyte-like cells [152]. Experiments using adipose tissue cultures from obese horses with insulin resistance and controls revealed that adding SHBG restored levels of INSR, INRS1/2, Akt and Pi3k, implying improved insulin sensitivity, and tended to normalize the fatty acid profile [153]. The addition of SHBG also increased ERK 1/2 phosphorylation in cultured human adipocytes in which lipolysis was increased [154].

A second potential SHBG-related mechanism for cellular transduction involves the endocytosis receptor megalin (LDL receptor-related protein 2) that plays a role in the endocytosis of 25OH vitamin D binding protein and other proteins and small molecules via the kidney proximal tubule [155]. Megalin is also expressed in prostate, epididymis, ovary and uterus, and choriocarcinoma cells. Global knock-out of megalin produced male mice with cryptorchidism and females with a closed vaginal opening [156] while prostate-specific knock-out resulted in lower levels of testosterone and dihydrotestosterone in the prostate [157].

Given the strong association between SHBG levels and MetS and T2DM, as well as evidence that genetic-raising alleles lower risk for these metabolic disorders [33,34], some authors have proposed a role for SHBG in the pathogenesis of IR and T2DM [158]. Data from the UK Biobank, with samples from >400,000 men and women, have shown that SHBG-lowering alleles are associated with an increased risk for T2DM in men and women, and for PCOS [77].

A series of experiments have been conducted to examine effects of SHBG using a mouse model in which the human SHBG transgene is over-expressed. These mice have circulating mM levels of human SHBG but also testosterone levels that are 10–100 times normal. They exhibit no phenotypic abnormalities, however, and males and females have normal reproductive function [159]. Cross breeding SHBG transgenic mice with db-db mice, that lack the leptin receptor, reduced the extreme weight gain and hepatic fat accumulation that typifies male db-db mice [160]. In a second series of experiments, high fat diet-induced fatty liver [11] as well as insulin and leptin levels [154] were markedly attenuated in the SHBG-transgenic male mice when compared to C57bl/6 controls. These results should be interpreted in light of the low testosterone levels that characterize both db-db mice [160] and normal C57bl/6 mice fed a high-fat diet [161] and the very high testosterone levels in SHBG transgenic mice [159]. Moreover, a second research group [162] reported no significant difference in body weight gain or percent body fat by dual-energy X-ray absorptiometry in the same SHBG-transgenic mice versus wild-type littermates fed a high-fat diet for 4.5 months rather than 8 wks. In the latter study, there was also no difference in fasting glucose or insulin levels or in glucose levels following glucose or insulin stimulation in the two groups [162]. It should be noted that these experiments included both males and females and the number of subjects per group was sometimes relatively small. Overall, these provocative but conflicting studies await confirmation, and the notion that SHBG exerts steroid-independent metabolic effects such that SHBG analogs might represent a novel treatment for metabolic disorders remains preliminary.

## 7. SHBG, a Biomarker of Metabolic Disease in Children and Adolescents

Childhood obesity remains a pressing public health concern, characterized by its widespread prevalence and strong links to chronic illnesses and reduced life expectancy [163]. Emerging research continues to reveal troubling rises in childhood obesity rates and its associated conditions, including MetS, MAFLD, T2DM, and PCOS in youth [163,164,165,166,167]. These conditions are interconnected, with multifactorial etiologies driven by genetic predispositions, the intrauterine environment and modern lifestyle changes, including unhealthy diets and physical inactivity [163,168,169]. Puberty, a developmental milestone characterized by significant hormonal, physiological, and metabolic shifts, plays a crucial role in the progression of these conditions [12,15,170].

Low circulating levels of SHBG represent a promising biomarker for obesity-related metabolic dysfunctions beginning in childhood. A growing body of evidence supports the inverse association between SHBG levels and obesity, IR, MetS, MAFLD, T2DM, and PCOS in youth [10,104,171,172]. Additionally, neither meal consumption nor diurnal variation appears to influence SHBG levels and SHBG can be measured with minimally invasive techniques such as a finger-stick blood sample [3].

SHBG may function during normal childhood to restrict the actions of sex steroids until puberty. In support of this hypothesis, a homozygous SHBG variant (rs6258) associated with a very low level of SHBG in serum was reported in a young girl with gonadotropin-independent precocious puberty [27]. SHBG levels decline in late childhood and during puberty in both sexes resulting in a progressive increase in free- when compared to total- sex hormone levels [52,53,54]. The underlying cause of the decline in SHBG remains unclear, but it is thought to be primarily metabolic [3,53], rather than hormonal, as age-related reductions are also observed in boys with hypopituitarism [56].

Insulin sensitivity decreases and insulin secretion increases in both girls and boys during puberty [12,173], potentially contributing to reduced SHBG levels. In a study of 132 healthy children and adolescents, SHBG was strongly linked to insulin sensitivity, even after accounting for puberty, fat mass, and aerobic fitness [73]. Researchers found a negative association between SHBG levels and metabolic risk, proposing that SHBG may reflect the interplay between changes in glucose metabolism and body composition during puberty. They also suggested its potential use as a marker for CVD risk during puberty [73].

Pinkney et al. [53] found that lower SHBG concentrations at 5 years of age predicted earlier puberty milestones in girls; SHBG was inversely correlated with markers such as body fat, insulin, IGF-I, CRP, and leptin. Similarly, girls diagnosed with central precocious puberty had reduced SHBG levels compared with age-matched healthy peers. Furthermore, the decline in SHBG with increasing age from 9 to 13 years in normal boys and girls was associated with increasing fat mass [174].

Studies show lower SHBG levels in boys with obesity versus normal-weight peers [106,175]. Pubertal boys with obesity also typically have lower total testosterone levels than normal-weight boys at the same stage of pubertal development, which is likely due, at least in part, to the lower levels of SHBG [91,176,177]. Pinkney et al. [53] also observed that boys with lower SHBG concentrations at age 5 tended to enter Tanner stage 2 earlier; however, no association was found between SHBG levels and either the timing of LH secretion onset or the age at peak height velocity.

Similar to adults, children and adolescents with MetS also exhibit reduced SHBG levels [9,82,107]. In a study of 815 Spanish school children, de Oya et al. [9] found that adolescents diagnosed with MetS or presenting features such as central obesity, elevated blood pressure, increased insulin or low HDL-C, had lower SHBG levels. Agirbasli et al. [82] also showed that low SHBG significantly predicted low HDL-C levels in Turkish youth. An in-depth metabolic analysis of 6475 young adults from two Finnish population-based cohorts demonstrated a link between SHBG and circulating lipids as well as metabolites related to adiposity and IR [178]. Recently, a study by Urbano et al. [10] found that adolescents with PCOS and MAFLD had lower SHBG levels and other CVD risk factors, such as high blood pressure and hyperinsulinemia and increased IR.

Moreover, SHBG holds promise as an important biomarker for MetS risk in children and adolescents well before the condition fully develops. Glueck et al. [172] reported that low SHBG levels in 14-year-old U.S. schoolgirls were more common in girls who developed MetS a decade later. Additionally, Wang et al. [178] showed that low SHBG levels among young adult Finnish men and women were predictive of IR development by HOMA-IR 6 years later, and these associations remained significant after adjusting for baseline adiposity, insulin and testosterone levels. Thus, there is evidence to support the idea that measuring SHBG levels can provide insights into a child’s metabolic health, particularly during puberty when traditional markers may be less predictive.

**Figure 5:**
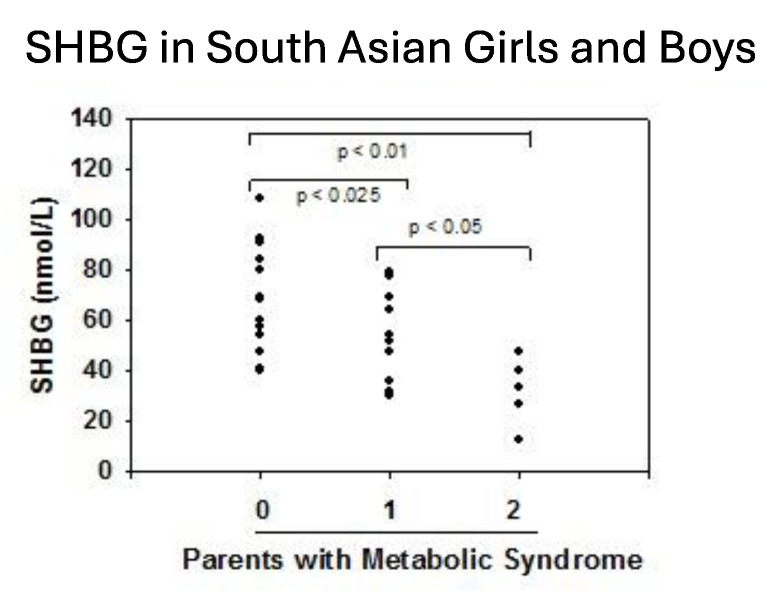
Serum levels of SHBG in South Asian-Indian children according to the diagnosis of metabolic syndrome in their parents. Redrawn from Krishnasamy et al. [8].

Prior studies have suggested a genetic predisposition to MetS [179,180] with ethnic variations in the distribution of MetS components in children [181,182,183,184]. As discussed above, SHBG levels vary by ethnicity. In the NHANES study, Mexican-American males aged 12–19 exhibited lower SHBG levels than their non-Hispanic black and white counterparts [96]. Abdelrahaman et al. [91] found that healthy African-American prepubertal boys tend to have higher SHBG levels, a group characterized by greater subcutaneous but lower VAT compared to white peers. South Asians are an ethnic group with a particularly high susceptibility to MetS and T2DM despite often having a low BMI. Research by Krishnasamy et al. [8] revealed that prepubertal South Asian-Indian children with one parent diagnosed with MetS exhibited 24% lower SHBG levels, while those with both parents affected showed 55% lower SHBG levels (Figure 5). The study also identified an inverse correlation between SHBG levels and both waist circumference and BMI percentile in these children. Furthermore, significant associations were found between SHBG (rs6257), cholesterol ester transfer protein (rs708272) polymorphisms and high triglycerides, low HDL-C and high LDL-C levels in a cohort of 365 Turkish youths [185]. White et al. [38] showed that SNPs within the SHBG gene (rs1799941) were linked to MetS in children. This association persisted after adjusting for individual MetS components, suggesting that no single factor was driving the link. While the A allele of rs1799941 was linked to higher SHBG levels in healthy controls, this effect was not observed in children with MetS.

## 8. Conclusions

MetS poses a significant health challenge, not only in adults but increasingly among adolescents. SHBG, a hepatic glycoprotein that transports testosterone and other steroids in the circulation, has emerged as a promising biomarker for assessing MetS risk due to its strong associations with key metabolic indicators such as IR, adiposity, and dyslipidemia. The decline in SHBG levels as puberty approaches, and its link with metabolic risk, highlights the potential importance of SHBG as an early screening tool, but more research is needed.

Racial and ethnic variations in SHBG levels emphasize the need for personalized reference ranges to enhance early detection efforts. Real-world applications of SHBG measurement may include risk stratification and early identification of youth at risk for CVD and additional criteria for the diagnosis of endocrine disorders such as PCOS.

While current research reveals an association between SHBG and metabolic health, further studies are needed to explore its regulatory mechanisms, role as a biomarker, and therapeutic potential of SHBG and related proteins. Early identification of children at risk through SHBG measurements could pave the way for targeted interventions and ultimately reduce the long-term burden of MetS and its associated complications.

## Figures and Tables

**Table 1:** Factors that influence the level of SHBG in blood.

Decrease	Increase
Androgens	Estrogens
Obesity	Pregnancy
Insulin resistance	SERMs *
Metabolic syndrome	Thinness
Type 2 diabetes mellitus	Weight loss
Gestational diabetes mellitus	Alcoholic liver disease
Polycystic ovary syndrome	Hepatitis-B and hepatitis-C infection
Metabolic fatty liver disease	Hyperthyroidism
Acromegaly	Thyroid hormone receptor-b agonists
Cushing syndrome	Growth hormone deficiency
Congenital adrenal hyperplasia	Hemochromatosis
Hyperprolactinemia	Acute intermittent porphyria
Tumor necrosis factor alpha	First generation anticonvulsants
Interleukin-1 beta	Mitotane
Genetic polymorphisms	Genetic polymorphisms

* Selective Estrogen Receptor Modulators (SERMs).

## Data Availability

Data sharing is not applicable to this article.
